# Differential analysis of mutations in the Jewish population and their implications for diseases

**DOI:** 10.1017/S0016672317000015

**Published:** 2017-05-15

**Authors:** YARON EINHORN, DAPHNA WEISSGLAS-VOLKOV, SHAI CARMI, HARRY OSTRER, EITAN FRIEDMAN, NOAM SHOMRON

**Affiliations:** 1 Faculty of Medicine, Tel Aviv University, Tel Aviv, Israel; 2 Braun School of Public Health and Community Medicine, The Hebrew University of Jerusalem, Jerusalem, Israel; 3 Department of Pathology, Albert Einstein College of Medicine, Bronx, NY, USA; 4 Susanne Levy Gertner Oncogenetics Unit, Sheba Medical Center, Tel-Hashomer, Israel

## Abstract

Sequencing large cohorts of ethnically homogeneous individuals yields genetic insights with implications for the entire population rather than a single individual. In order to evaluate the genetic basis of certain diseases encountered at high frequency in the Ashkenazi Jewish population (AJP), as well as to improve variant annotation among the AJP, we examined the entire exome, focusing on specific genes with known clinical implications in 128 Ashkenazi Jews and compared these data to other non-Jewish populations (European, African, South Asian and East Asian). We targeted American College of Medical Genetics incidental finding recommended genes and the Catalogue of Somatic Mutations in Cancer (COSMIC) germline cancer-related genes. We identified previously known disease-causing variants and discovered potentially deleterious variants in known disease-causing genes that are population specific or substantially more prevalent in the AJP, such as in the *ATP* and *HGFAC* genes associated with colorectal cancer and pancreatic cancer, respectively. Additionally, we tested the advantage of utilizing the database of the AJP when assigning pathogenicity to rare variants of independent whole-exome sequencing data of 49 Ashkenazi Jew early-onset breast cancer (BC) patients. Importantly, population-based filtering using our AJP database enabled a reduction in the number of potential causal variants in the BC cohort by 36%. Taken together, population-specific sequencing of the AJP offers valuable, clinically applicable information and improves AJP filter annotation.

## 1. Introduction

High-throughput sequencing, also known as next-generation sequencing (NGS), reduced the cost and increased the yield of DNA sequencing. As whole-exome sequencing (WES) and whole-genome sequencing (WGS) are increasingly integrated into practical medical care, the importance of studying the genetic structure of ethnically diverse populations using NGS rises. Although most of the variant sites in the human genome are shared among individuals, allele frequencies vary substantially between populations (The International HapMap Consortium, 2005; 1000 Genomes Project Consortium *et al*., 2012; Visscher *et al.*, 2012; Carmi *et al.*, 2014; The Genome of the Netherlands Consortium, 2014; Gudbjartsson *et al.*, 2015; Nagasaki *et al.*, 2015). The value and advantages of sequencing diverse populations has already been shown in: genome-wide association studies (Visscher *et al.*, 2012); discovering rare and *de novo* variants; improving variant calling sensitivity and specificity; and improving the accuracy of curating pathogenic variants (Carmi *et al.*, 2014; The Genome of the Netherlands Consortium, 2014; Gudbjartsson *et al.*, 2015). Substantial efforts have been devoted to sequencing large number of individuals from diverse populations in order to create public databases that can assist human genetic studies such as the 1000 Genomes Project (1KG) (1000 Genomes Project Consortium *et al*., 2012), the Exome Sequencing Project (ESP; http://evs.gs.washington.edu/EVS/) and the Exome Aggregation Consortium (ExAC; http://exac.broadinstitute.org/).

The Ashkenazi Jewish population (AJP) is known to have a high rate of several diseases affecting individuals of that ethnic origin compared with other world ethnicities (Rosner *et al.*, 2009). These include both autosomal recessive disorders due to the founder effect (Slatkin, 2004; Bray *et al*., 2010; Carmi *et al.*, 2014), such as Gaucher disease (Beutler *et al.*, 1993), cystic fibrosis (Abeliovich *et al.*, 1992) and Tay–Sachs disease (Myerowitz & Costigan, 1988), as well as more common, adult-onset autosomal dominant diseases such as Parkinson's disease (PD) (Ozelius *et al.*, 2006) and hereditary BC and ovarian cancer (Struewing *et al.*, 1997). Notably, the AJP has not been included as part of large-scale international sequencing projects. A recent NGS study of an AJP cohort demonstrated an improvement in imputation accuracy and modelling of Jewish history (Carmi *et al.*, 2014). However, further research is warranted in order to elucidate the possible clinical implications of the AJP allelic architecture and to improve the curation and accuracy of pathogenic variant screening in current and future AJP studies.

Recently, new recommendations for the AJP screening panel were published based on the same dataset as ours (Baskovich *et al.*, 2016). However, that study focused only on the identification of pathogenic variants for the purpose of clinical screening in the AJP, whereas the current study takes a more global view by focusing on the genome and gene-level trends, rather than particular genetic variants, examining the utility of using an AJP-specific reference panel in interpreting clinical sequencing projects involving AJP individuals.

In this study, we focused on the clinical utility and practical implications resulting from WES analysis of 128 Ashkenazi Jews, of whom 74 individuals had no discernible disease and 54 were controls in a PD study. We examined the genetic differences between the AJP and other non-Jewish populations (NJPs) and searched for genes that are more likely to carry pathogenic variants among the AJP than in NJPs. Finally, we applied our findings to 49 independent Ashkenazi Jewish BC patients in order to evaluate the value of utilising an Ashkenazi Jew-specific database as a filtering tool.

## 2. Methods

### Ashkenazi Jew variants

We used an unfiltered variant calling file (VCF) of 128 verified Ashkenazi Jewish individuals who underwent WGS as a part of a population genetic study of the AJP (Carmi *et al.*, 2014). WGS was conducted by Complete Genomics with a high coverage (average coverage >50×). Seventy-four of the individuals were considered healthy and 54 were controls in a PD study. We extracted variants from the whole-exome region only, based on Ilumina's TruSeq Exome Enrichment Kit targets (https://www.illumina.com/content/dam/illumina-marketing/documents/products/datasheets/truseq-exome-data-sheet-770-2015-007.pdf), and did not include areas outside this region in our bioinformatics analysis. The target region size was 62 Mb, which targets 20,794 genes and 96·4% of RefSeq43-coding exons. We performed quality check (QC) and applied different filtrations (see Supplementary Methods; available online), which resulted in 222,179 high-quality single-nucleotide variants (SNVs).

### BC patient variants

The VCF of 49 Ashkenazi Jewish BC patients, suspected to be hereditary, was obtained using the Genome Analysis Toolkit (GATK) best practice pipeline (McKenna *et al.* 2010), followed by QC (see Supplementary Methods), which resulted in 173,300 variants for the same exome region as the 128 Ashkenazi Jews.

### 1KG control groups

As control groups, and in order to compare the AJP with other populations, we used the European, African, East Asian (EAS) and South Asian (SAS) populations from the 1KG Project version 3 database (1000 Genomes Project Consortium *et al*., 2012). The data for these datasets were generated using the Illumina platform, and the variants were called by combining different variant callers, among them GATK's variant caller (http://www.1000genomes.org/analysis). For each population, 128 individuals were selected randomly, and the same region that was examined for the AJP was extracted.

## 3. Results

**Fig. 1.**
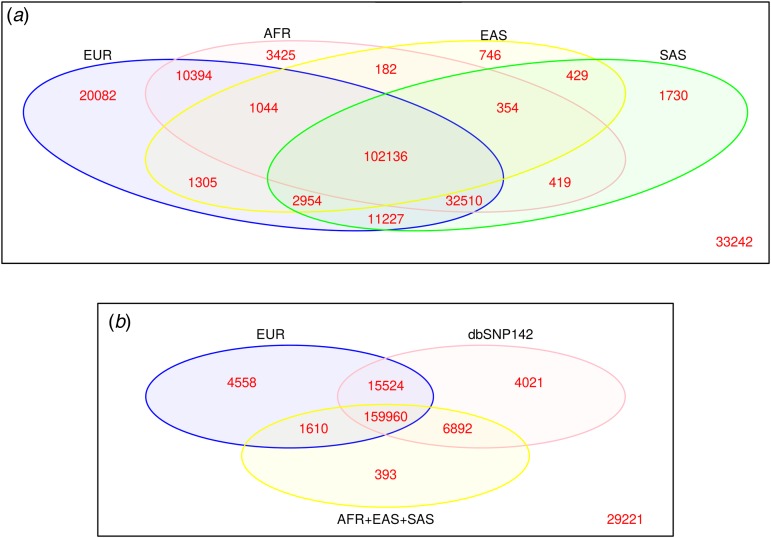
(*a*) Overlap of the Ashkenazi Jewish population (AJP) variants with the European (EUR), African (AFR), East Asian (EAS) and South Asian (SAS) populations' variants. (*b*) Only 3·2% of the variants overlap with one of the non-EUR distal populations. Crossing with the dbSNP142 database resulted in 29,221 novel variants unique to the AJP.

In this study, we analysed the whole-exome data of 128 Ashkenazi Jewish individuals. We detected 222,179 SNVs, of which 30·6% (68,139) were singletons and 81·7% were shared and were annotated in other European population databases, including the European samples of ESP, ExAC and 1KG. Although this rate of overlap between the AJP and the European population is in line with the known relatedness and genetic similarity between the European population and the AJP (Behar *et al.*, 2003; Costa *et al.*, 2013), approximately 20% of the detected variants were unique to the AJP. The overlap rates between AJP variation and genetically more distant populations including African, EAS and SAS populations (inferred from ExAC and 1KG databases) were significantly smaller, as expected (68–49%, Fig. 1(*a*)), further strengthening the validity of our data. Only 3·2% of the AJP variants were present in one of these distantly related populations but not in the European dataset, resulting in 13·3% (29,221) AJP-unique (i.e. novel) variants not reported in any of the population databases or in dbSNP142 (Fig. 1(*b*)).

Next, we functionally annotated the coding variants and classified the exonic variants into three categories by severity: (i) ‘high impact’ including stop-gain or stop-loss variants and variants within 2-bp of a splicing junction; (ii) ‘moderate impact’ included exonic missense variants; and (iii) ‘low impact’ included synonymous variants and exonic variants of unknown type due to incomplete gene structure information. Using this classification scheme, 831 variants (19 splice site variants) with high impact were identified, 54,585 were moderate-impact variants and 45,876 were low-impact variants. A similar distribution of variant severity was observed in the 128 European individuals (Supplementary Fig. S1).

### Evaluating ACMG and COSMIC set of genes

We evaluated the clinical implications of the high-impact, very rare variants by comparing the existence of these variants in two gene sets: the Catalogue of Somatic Mutations in Cancer (COSMIC; http://cancer.sanger.ac.uk/cosmic) and the American College of Medical Genetics and Genomics (ACMG; https://www.acmg.net/). COSMIC's Cancer Genes Census catalogues genes that exhibit mutations that are causally implicated in cancer pathogenesis (see Supplementary Material for the complete list). Of all COSMIC genes harbouring germline cancer mutations (*n* = 87) associated with cancer predisposition, six high-impact variants in five cancer predisposition genes were noted (Supplementary Table S2). Five of the variants were singletons, and one was a doubleton: rs34295337 in *ERCC3*, a gene associated with xeroderma pigmentosum type B (Ma *et al.*, 1994), which is a rare autosomal recessive disease that is associated with skin cancer (Paszkowska-Szczur *et al.*, 2013). One variant, rs11571833, in the *BRCA2* gene, was described previously as being associated with an increased risk of developing a variety of cancer types including lung, breast, prostate, gastric and aerodigestive tract cancer (Wang *et al.*, 2014; Delahaye-Sourdeix *et al.*, 2015; Thompson *et al.*, 2015; Meeks *et al.*, 2016; Vijai *et al.*, 2016). Two variants, one in the *DICER1* gene and one in the *NF1* gene, were novel. The *NF1* gene harboured one additional high-impact variant. Notably, *NF1* germline mutations underlie the neurofibromatosis type 1 phenotype, a disease that is reportedly diagnosed at higher rates in the AJP than in the European population (Garty *et al.*, 1994).

The ACMG recommendation for reporting incidental findings in clinical sequencing includes 56 genes (22 genes intersect with COSMIC genes; see Supplementary Material for the complete list). High-impact variants were noted in two ACMG genes. The first variant (rs11571833) in the *BRCA2* gene was already described and discussed above. The second variant, rs200563280, results in a premature stop codon in the *RYR1* gene, a gene that is associated with malignant hyperthermia (Robinson *et al.*, 2006). Thus, the rate of actionable incidental findings in the AJP is 1·56%, similar to the estimate for Europeans at approximately 2% (Amendola *et al.*, 2015). None of the above variants were mentioned in a recent study, based on the same dataset that expanded the recommendations for an AJP screening panel (Baskovich *et al.*, 2016).

### AJP-specific variants

We next examined AJP-specific variants. We defined variants as AJP specific if they were unique (i.e. novel) or very rare (minor allele frequency (MAF) <1%) in the NJPs, but more prevalent in the AJP (MAF >1%). Of the total AJP variants, 17,977 (8%) were AJP specific. To confirm that our dataset is enriched with variants that are unique to the AJP, we performed the same analysis on 128 verified Europeans from the Personal Genome Project (PGP) (Church, 2005; see Supplementary methods). Only 8748 variants (3·6% of the PGP dataset) were more than 1% in the PGP dataset but not in NJPs (both European and non-European populations).

**Fig. 2.**
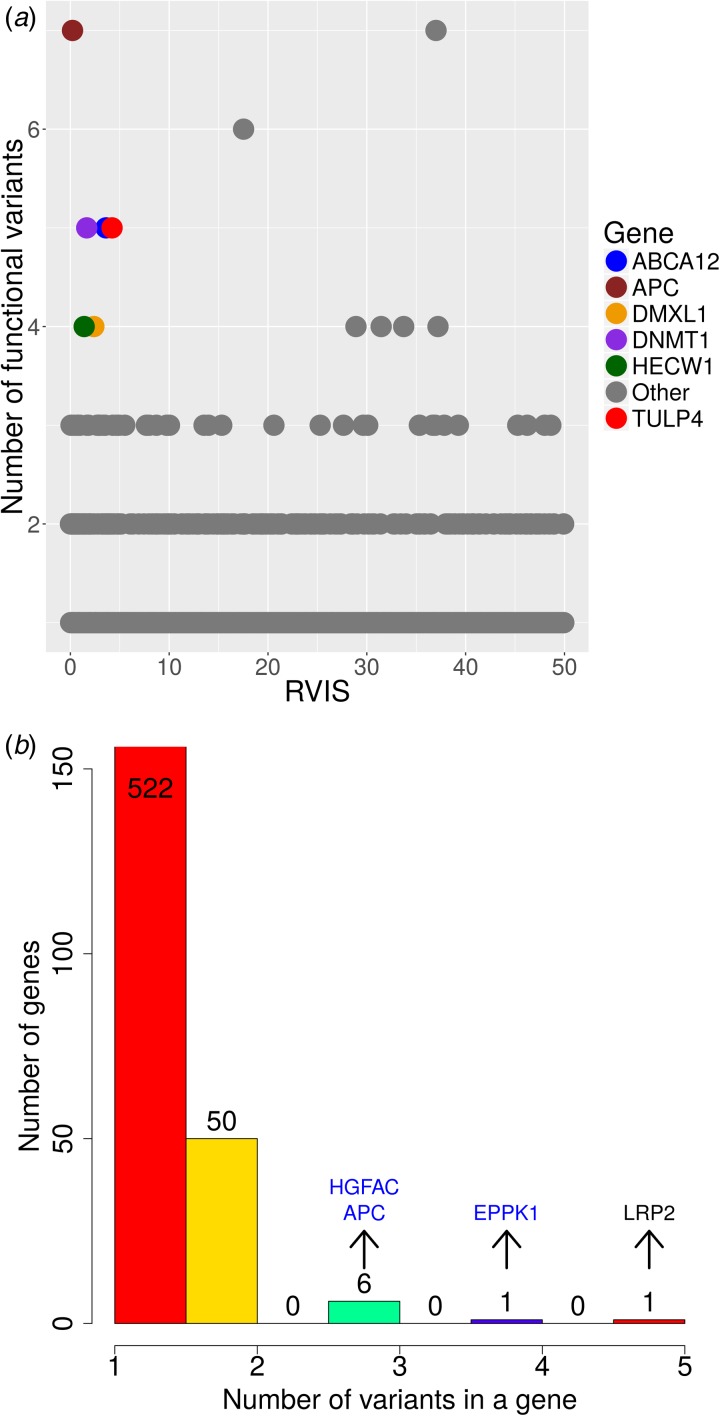
(*a*) Genes with a low residual variation intolerance score (RVIS) are less tolerant to rare functional variants. Only six genes had a very low RVIS and four or more high to moderate Ashkenazi Jewish population (AJP)-specific variants, including the *APC* gene, which had the lowest RVIS and highest number of variants at seven. (*b*) Histogram of the number of AJP-specific deleterious variants in a gene. While most of the genes had two or fewer of these variants, eight genes had three to five variants.

We then looked at genes that are enriched for moderate- to high-impact variant groups that are AJP specific. This analysis yielded 5142 variants. Most genes harboured up to one such variant, 840 genes exhibited two variants and 196 genes displayed three or more moderate- to high-impact variants (Supplementary Fig. S2). After QC (see Supplementary Methods), three outlier genes were filtered out (Supplementary Fig. S2). In this analysis, virtually no correlation between the number of variants and the genomic length of the gene was observed (Pearson's correlation = 0·1). Next, we examined the residual variation intolerance score (RVIS) (Petrovski *et al.*, 2013) in order to identify genes under purifying selection that harbour unique or prevalent mutations in the AJP. Briefly, RVIS measures the tolerance of a gene to contain damaging variation. Genes with a low RVIS are predicted to be less tolerant to variation, and hence are more likely to exhibit a phenotype due to non-synonymous variants. The *APC* gene harboured a high number of AJP-specific variants (*n* = 7) and is in the lowest 0·2 percentile of RVIS (Fig. 2(*a*)). Mutations in the *APC* gene are associated with a specific form of inherited predisposition to colorectal cancer. Overall, colorectal cancer is more prevalent in the AJP than in NJPs (Feldman, 2001). Notably, the p.I1307K missense mutation in *APC* (rs1801155), which has been previously shown to moderately increase colorectal cancer risk in the AJP (Woodage *et al.*, 1998), was among the identified variants (MAF = 0·047), and was recommended for inclusion in AJP screening (Baskovich *et al.*, 2016). However, additional susceptibility variants were detected in the *APC* gene, suggesting that other variants may contribute to the increased prevalence of colorectal cancer in the AJP. Other genes with low RVIS and harbouring four AJP-specific damaging variants are *ABCA12, TULP4, DNMT1, DMXL1* and *HECW1*. To the best of our knowledge, the prevalence of the phenotypes associated with these genes (Supplementary Table S3) is not significantly higher in the AJP compared with other NJPs. Hence, the clinical implications and significance of this seemingly high rate of damaging variants in these genes warrant further investigation in additional extended Ashkenazi Jewish studies.

To assess the effect of the AJP-specific variants on protein function, we used the MetaLR (Dong *et al.*, 2015) ensemble tool, which integrates different prediction tools using logistic regression to predict whether a variant is deleterious (see Supplementary Methods). Overall, we obtained 649 AJP-specific deleterious variants in 580 different genes. Only eight genes had at least three AJP-specific deleterious variants (Fig. 2(*b*) and Supplementary Table S3): *APC, ABCA12, LRP2, EPPK1, HGFAC, ACAD11, HLCS* and *NOX1. APC* and *ABCA12* were discussed; the *HGFAC* (three variants) gene is a member of the peptidase S1 protein family and is associated with pancreatic cancer (Kitajima *et al.*, 2008), a cancer type that is known to be more frequent among the AJP (Feldman, 2001). The *EPPK1* gene (four variants) encodes a protein that belongs to the plakin family and is related to ‘vacterl association’ disorder (Hilger *et al.*, 2013). The phenotype of this disorder encompasses Fanconi anaemia, a phenotype that is diagnosed at a higher frequency in the AJP compared with NJPs (Kutler & Auerbach, 2004), and hence, these variants may contribute to these higher occurrence rates. The other genes are associated with different types of rare diseases, but to the best of our knowledge, these conditions are not diagnosed at an increased rate in the AJP (Supplementary Table S3).

Furthermore, to examine whether the genes harbouring AJP-specific deleterious variants were previously implicated as AJP-prevalent phenotypes, we queried VarElect (http://varelect.genecards.org/) using the term ‘Ashkenazi’. VarElect can prioritise genotype–phenotype associations based on various databases. Of the 580 queried genes, 14 genes harbouring 17 variants (Table 1) were found to be directly related to the ‘Ashkenazi’ term, denoting conditions that are common to the AJP. Five of the 17 variants are considered to be pathogenic by the Clinvar database, four of the variants were also included in the recent recommendation for the AJP screening panel (Baskovich *et al.*, 2016) and four of the genes are included in the AJP screening panel, but for different variants. To verify our results, we did the same for the 128 European individuals looking at European-specific variants, meaning genes with variants that were very rare in the non-European population but not in the European population (423 genes), and tried to find genes that were related to the ‘Ashkenazi’ phenotype. Although 20 genes were found to be related, none of the variants in them was found to be pathogenic by Clinvar, which further supports our results. Taken together, these results suggest that additional variants, among these 17 variants, are plausibly causal and hence should be further investigated.

**Table 1.** Ashkenazi Jewish population-specific deleterious predicted variants in genes that relate to Ashkenazi Jews according to VarElect.

Gene	Number of AJP-specific deleterious variants	rs number	RVIS percentile	VarElect score^*a*^	Diseases related to gene by VarElect^*b*^	Diseases related to variant by Clinvar^*c*^	Gene/variant mentioned in Baskovich *et al*. (2016)^*d*^
*APC*	3	rs140493115, chr5:112175676, rs200756935	0.22	5.36	Colorectal cancer	−	Gene
*WFS1*	2	rs71524353, rs35218685	98.91	1.39	Type 2 diabetes	−	−
*AK3*	1	rs141090350	26.48	1.39	Crohn's disease	−	−
*ASPA*	1	rs28940279	63.46	2.4	Canavan disease	Canavan disease	Variant
*F11*	1	rs121965064	14.91	3.1	Factor XI deficiency	Factor XI deficiency	Variant
*COL4A3*	1	rs55816283	48.42	1.39	Alport syndrome	−	−
*CFTR*	1	rs1800073	81.9	1.39	Cystic fibrosis	−	Gene
*DLD*	1	rs121964990	20.72	2.77	Lipoamide dehydrogenase deficiency	Dihydrolipoyl dehydrogenase deficiency	Variant
*LDLR*	1	rs137853964	9.94	1.96	Familial hypercholesterolaemia	Familial hypercholesterolaemia	Gene
*NDUFS4*	1	chr5:52942219	44.18	1.39	Leigh syndrome	−	Gene
*NOD2*	1	rs104895438	91.35	3.39	Crohn's disease	Sarcoidosis	−
*KCNH6*	1	rs139719681	92.33	1.39	Crohn's disease	−	−
*TIMP1*	1	rs149986790	94.82	1.39	Refractive error	−	−
*VKORC1*	1	rs61742245	88.61	1.96	Warfarin pharmacogenetics	Warfarin pharmacogenetics	Variant

a A score given by VarElect to show how much the gene was found to be related to the Ashkenazi phenotype.

b The diseases that were related to this gene in the context of the Ashkenazi phenotype according to VarElect.

c Diseases that were related to the variant according to Clinvar.

d Is the gene or the variant also found in a recent recommended AJP screening panel?

AJP = Ashkenazi Jewish population; RVIS = residual variation intolerance score.

### Using the Ashkenazi Jewish database in an analysis of Ashkenazi Jewish early BC patients

**Fig. 3.**
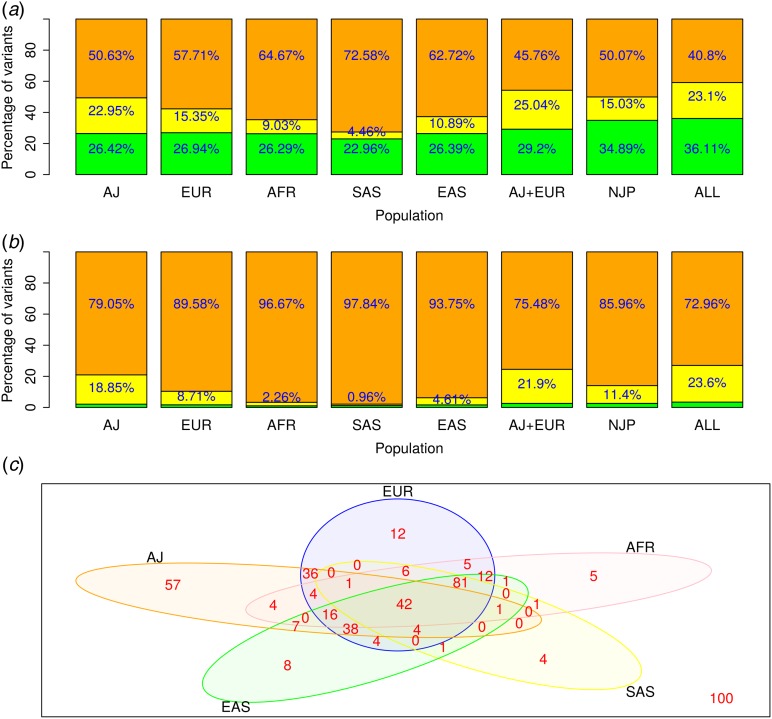
Frequencies of high- to moderate-impact variants (*a*) and deleterious variants (*b*) by populations' minor allele frequency (MAF) (orange – very rare; yellow – rare; green – common). By joining the Ashkenazi Jewish population (AJP) MAF to the non-Jewish population (NJP) MAF and using the maximum MAF, the percentages of very rare variants were reduced by 10% and 13%, respectively. (*c*) Filtration of very rare variants of 49 Ashkenazi Jewish (AJ) breast cancer patients. Adding the AJ MAF filtered an additional 57 (36%) of the variants, demonstrating the utility of using the same population database. AFR = African; EAS = East Asian; EUR = European; SAS = South Asian.

The major objective of clinical sequencing is to identify the causative mutation from amongst numerous detected variants. To that end, non-synonymous variants with rare allele frequencies are considered initially as plausible causative mutations. Since the AJP is not included in any of the public databases of international sequencing efforts, the MAFs of closely related populations such as Europeans (Haas *et al.*, 2012; Lee *et al.*, 2012; Rees *et al.*, 2012) are often utilised as surrogates. We evaluated the advantages of using AJP-specific MAFs when screening the WES data of Ashkenazi Jewish samples. Of the 55,416 high- and moderate-impact mutations, 57·7% were classified as very rare based on the general European MAF versus 50·6% based on the AJP MAF, leading to out-filtration of approximately 3900 variants (Fig. 3(*a*)). Likewise, based on the maximum MAF (MMAF) of all NJPs, 50·1% of the variants were classified as very rare, compared to 40·8% when including the AJP. These results are in line with Carmi *et al.* (2014). For rare variants (MAF <5%), the advantage of using AJP-specific MAFs is somewhat less significant (1·2% difference), in line with the notion that population-specific variants are predominantly very rare (1000 Genomes Project Consortium *et al*., 2015).

Similarly, potentially deleterious variants are prioritised in clinical NGS applications. Based on the AJP MAF, 79·0% of deleterious variants, based on MetaLR, were considered very rare, whereas 89·6% were considered very rare based on the European MAF (Fig. 3(*b*)). Furthermore, combining the AJP MAF with the NJP MMAF substantially improved filtering from 85·9% of the variants classified as very rare to just 72·9%. Since the MAFs of numerous populations, but not the AJP, are included in the MetaLR model, adding the AJP MAF can significantly improve the filtering of deleterious variants. Taken together, these significant population-specific differences in rare variants indicate that by utilising AJP-specific MAFs, finer filtration and lower false-positive rates can be achieved in Ashkenazi Jewish sequencing studies.

Importantly, we evaluated the utility of the AJP-specific screening approach using the independent WES data of 49 Ashkenazi Jewish samples derived from high-risk BC cases who do not harbour mutations in the predominant underlying genes – *BRCA1* and *BRCA2*. Of the 2638 predicted deleterious variants, 81·3% were very rare according to the European MAF, compared to 77·5% using the AJP MAF. Similarly, combining the AJP with the NJP MMAF improved filtering by approximately 10% from 75·9% to 64·5% (Supplementary Fig. S3).

In our actual disease gene analysis of the Ashkenazi Jewish BC sample, we screened for very rare variants that are potentially deleterious by MetaLR and are present in at least three BC cases, resulting in 450 potentially deleterious variants. Filtering by using the European MAF resulted in 189 variants in 148 genes, while using the MMAF of the Ashkenazi Jewish and Europeans filtered an additional 69 variants, resulting in 120 potential variants (36%). In comparison, using the MMAF of Europeans and 128 individuals from African, EAS or SAS populations resulted in minor additional filtering of only seven, two and 13 variants, respectively (Supplementary Fig. S4). Using all populations' MMAFs (AJP + NJP) versus only the NJP MMAF resulted in 100 variants in 72 genes compared to 157 variants in 126 genes (36%) (Fig. 3(*c*)). We then used VarElect to search for genes related to the keyword ‘breast’. The *MSH6* gene scored highest using VarElect (Supplementary Table S4) and by the MetaLR deleterious score (0·88). The protein coded by this gene is a member of the DNA mismatch repair MutS family, and rare variants in this gene are associated with familial BC (Wasielewski *et al.*, 2010). Mutations in *MSH6* are traditionally associated with Lynch syndrome (Baglietto *et al.*, 2010), a syndrome that seems to encompass BC susceptibility according to recent publications (Win *et al.*, 2013). This finding requires further examination of a larger cohort in order to draw better conclusions about the role of these variants in BC predisposition.

## 4. Discussion

In this study, a comprehensive analysis of the whole exome in 128 Ashkenazi Jewish individuals using high-coverage NGS technology was carried out and compared with the same data generated from a closely related European population.

By targeting AJP-specific variants, the clinical utility of using NGS technology to genotype entire populations is clearly demonstrated. Using such an approach, applying a variety of bioinformatics and predictive tools and querying several publicly available databases, we revealed novel variants and genes that may be associated with an increased risk of developing a host of diseases in the AJP. Some of these variants occur within genes related to diseases that are known to be more commonly diagnosed in the AJP than in NJPs: colorectal cancer (*APC* gene) and pancreatic cancer (*HGFAC* gene). Although these variants are predicted to be pathogenic and may indeed affect cancer risk, the current evidence is still tentative and cannot be clinically applied until validation and expansion of these results is provided by future studies. The *EPPK1* gene harboured a few AJP-specific deleterious variants. Homozygous mutations in this gene are associated with Fanconi anaemia, a disorder that is more commonly encountered in AJP (Kutler & Auerbach, 2004). Moreover, heterozygous mutations in Fanconi anaemia genes are associated with increased cancer risk, primarily BC (Mathew, 2006; Alan & D'Andrea, 2010), and indeed, two of the four AJP-specific deleterious variants in the *EPPK1* gene were also detected in the high-risk BC cohort. Among the observed AJP-specific deleterious variants, five were known to be pathogenic variants that increase the risk of five different diseases that are common to the AJP, and three of them were included in a new recommended screening panel for the AJP (Baskovich *et al.*, 2016). These overlaps confirm the effectiveness of the methodology applied in the present study for finding population-based pathogenic variants, as well as supporting the potential of population screening using NGS. Additionally, by examining specific genes with known and valuable clinical implications and consequences (i.e. ACMG incidental findings genes and COSMIC germline mutation-harbouring genes), a number of variants were identified in genes that lead to a phenotype that is seen at a higher occurrence in the AJP than in other populations (e.g. the *NF1* gene).

Based on the results of the present study and the current ACMG incidental findings recommendations, in approximately 3/200 (1·56%) members of the AJP who undergo WES, an incidental finding will emerge. As information about the role of each variant in the exome/genome accumulates and the pathogenicity prediction tools and functional analyses continue to evolve, some of the moderate-impact variants of these genes might also be reclassified as pathogenic, so that the rate of incidental findings may still be altered.

The present study also illustrated the importance of using the Ashkenazi Jewish-specific database in the course of analysing the genetic basis of inherited cancer in the AJP. Using the dataset and analysis tools, the number of potential causal sequence variants underlying an inherited predisposition to BC was reduced by 36%. Such a filtering step is critical to defining a bona fide causal mutation. Therefore, this provides further support for the importance of creating and using a population-specific database when investigating the genetic basis of inherited diseases, rather than using genetically related but not identical populations.

While a recent study of 5685 Ashkenazi Jewish exomes has been published (Rivas *et al.*, 2016), the current study provides evidence that by using whole-exome data from a relatively small number (*n* = 128) of Ashkenazi Jewish individuals, clinically relevant information and improvements in filter annotation are feasible. Thus, the research potential value and clinical benefits of using NGS technology at a population level are further emphasised.

## Supplementary material

For supplementary material accompanying this paper visit https://doi.org/10.1017/S0016672317000015. click here to view supplementary material
