# Supplementary material for: Differential analysis of mutations in the Jewish population and their implications for diseases
Source: Genet Res (Camb). 2017 May 15;99:e3. doi: 10.1017/S0016672317000015 (PMC6865140; doi:10.1017/S0016672317000015)
Supplement: Supplementary file 1 [file S0016672317000015sup001.docx]

**Differential analysis of mutations in the Jewish Population and its implications on diseases**

Yaron Einhorn^1^, Daphna Weissglas-Volkov^1^, Shai Carmi^2^, Harry Ostrer^3^, Eitan Friedman^1,4^, Noam Shomron^1^*

^1^Faculty of Medicine, Tel Aviv University, Tel Aviv, Israel

^2^Braun School of Public Health and Community Medicine, The Hebrew University of Jerusalem, Jerusalem, Israel

^3^Department of Pathology, Albert Einstein College of Medicine, Bronx, NY, USA

^4^Susanne Levy Gertner Oncogenetics Unit, Sheba Medical Center, Tel-Hashomer, Israel

* Correspondence: Noam Shomron TEL 972-36406594 FAX 972-36407432 [nshomron@post.tau.ac.il](mailto:nshomron@post.tau.ac.il)

# **Supplementary tables**

### **Supplementary Table S1: Average load of variants per an AJ individual compared to a European individual**

|  | **AJ** |  |  |  |  | **EUR** |  |  |  |
| --- | --- | --- | --- | --- | --- | --- | --- | --- | --- |
|  |  |  |  |  |  |  |  |  |  |
|  | **Very rare (<1%)** | **Rare(1-5%)** | **Common (>5%)** | **All** |  | **Very rare (<1%)** | **Rare(1-5%)** | **Common (>5%)** | **All** |
|  | **Mean (s.d.)** | **Mean (s.d.)** | **Mean (s.d.)** | **Mean (s.d.)** |  | **Mean (s.d.)** | **Mean (s.d.)** | **Mean (s.d.)** | **Mean (s.d.)** |
|  |  |  |  |  |  |  |  |  |  |
| **All variants*** | 898.2 (71.0) | 2246.1 (89.0) | 37,341 (411.9) | 40,485(451.4) |  | 870.9(100.4) | 2101.7(97.2) | 43,822.2 (547.9) | 46,794.8 (571.8) |
|  |  |  |  |  |  |  |  |  |  |
| **Low impact**  **heterozygous** | 169.8 (16.9) | 471.1 (29.5) | 5587.4 (103.6) | 6228.4 (115.3) |  | 177.0 (22.0) | 430.7 (28.1) | 6123.4 (115.5) | 6731.2 (126.0) |
| **Low impact**  **homozygous** | 0.29 (1.0) | 6.1 (3.8) | 3076.6 (67.4) | 3083.1 (68.8) |  | 0.36 (0.65) | 7.9 (4.1) | 3528.0 (82.0) | 3536.3 (82.9) |
|  |  |  |  |  |  |  |  |  |  |
| **Moderate impact**  **heterozygous** | 266.1 (27.8) | 521.7 (29.2) | 4308.0 (99.1) | 5096.0 (112.7) |  | 273.3 (29.5) | 489.1 (35.6) | 5052.8 (133.1) | 5815.3 (141.2) |
| **Moderate impact**  **homozygous** | 0.41 (1.0) | 6.6 (4.0) | 2400.5 (57.7) | 2407.5 ( 59.0) |  | 0.75 (1.1) | 9.0 (4.6) | 2896.7 (83.6) | 2906.5 (84.5) |
|  |  |  |  |  |  |  |  |  |  |
| **High impact**  **heterozygous** | 0 | 5.9 (2.5) | 34.3 (4.6) | 45.4 (5.7) |  | 5.1 (2.4) | 6.6 (2.3) | 40.4 (6.3) | 52.2 (7.3) |
| **High impact**  **homozygous** | 0 | 0.09 (0.3) | 15.2 (2.9) | 15.3 (2.9) |  | 0.04 (0.19) | 0.08 (0.28) | 20.6 (3.2) | 20.8 (3.2) |
|  |  |  |  |  |  |  |  |  |  |
| **Deleterious predicted**  **heterozygous** | 37.0 (7.5) | 34.4 (5.5) | 22.9 (4.3) | 94.4 (10.2) |  | 34.8 (7.1) | 25.5 (6.1) | 40.6 (14.8) | 101.0 (17.6) |
| **Deleterious predicted**  **homozygous** | 0.05 (0.22) | 0.3 (0.6) | 5.0 (1.8) | 5.4 (1.9) |  | 0.07 (0.34) | 0.23 (0.47) | 6.1 (2.2) | 6.4 (2.3) |
|  |  |  |  |  |  |  |  |  |  |
| **OMIM genes genes high and**  **moderate impact**  **heterozygous** | 60.0 (9.1) | 113.6 (12.6) | 861.6 (30.7) | 1035.3 (36.7) |  | 60.4 (8.9) | 102.2 (12.1) | 1000.2 (47.2) | 1162.9 (50.9) |
| **OMIM genes high and**  **moderate impact**  **homozygous** | 0.06 (0.3) | 1.4 (1.5) | 461.7 (19.6) | 463.2 (19.6) |  | 0.1 (0.3) | 1.8 (1.5) | 564.4 (31.3) | 566.4 (31.2) |

*All variants includes all variants in the region which we investigated including intronic variants.

### **Supplementary Table S2: Cosmic and ACMG genes high impact variants**

| **rs number** | **Gene** | **Mutation type** | **AJ MAF** | **NJ MAX MAF** | **EUR MAF** | **Related disease by COSMIC/ACMG** |
| --- | --- | --- | --- | --- | --- | --- |
| rs34295337 | ERCC3 | Stop gain | 0.0078 | 8.000e-04 | 0.0008 | Xeroderma pigmentosum (skin cancer ) |
| rs11571833 | BRCA2 | Stop gain | 0.0039 | 1.090e-02 | 0.0109 | Hereditary breast/ovarian cancer |
| chr14:95577671 | DICER1 | Stop gain | 0.0039 | 0.000e+00 | 0 | Familial pleuropulmonary blastoma or DICER1 syndrome |
| chr16:89805698 | FANCA | Splicing | 0.0039 | 1.508e-05 | 1.508e-05 | Fanconi anemia ( leukaemia) |
| chr17:29553697 | NF1 | Stop gain | 0.0039 | 1.502e-05 | 1.502e-05 | Neurofibromatosis type 1 |
| chr17:29657361 | NF1 | Stop gain | 0.0039 | 0.000e+00 | 0 | Neurofibromatosis type 1 |
| rs34295337 | RYR1 | Stop gain | 0.0039 | 0.0010 | 0.001 | Malignant hyperthermia susceptibility |

### **Supplementary Table S3: Genes with a high number of AJP-specific high to moderate impact variants and with low RVIS, or with a high number of AJP-specific deleterious variants**

| **Gene** | **Function and related diseases** | **RVIS Exac percentile (low preceintile means less likelihood to have variants)** | **Number of AJP-specific moderate to high variants** | **Number of AJP-specific deleterious variants** |
| --- | --- | --- | --- | --- |
| APC | Encodes a tumor suppressor protein that acts as an antagonist of the Wnt signaling pathway. Related to Colorectal cancer | 0.22 | 7 | 3 |
| ABCA12 | A member of the superfamily of ATP-binding cassette (ABC) transporters, and known to be related to “ichthyosis, congenital, autosomal recessive 4a” and “ichthyosis, autosomal recessive 4b” diseases. | 3.61 | 5 | 3 |
| TULP4 | Related to sequence-specific DNA binding transcription factor activity. | 4.22 | 5 | 1 |
| DNMT1 | Maintain patterns of methylated cytosine residues in the mammalian genome and is related to “neuropathy, hereditary sensory, type ie” and “cerebellar ataxia, deafness, and narcolepsy, autosomal dominant” diseases. | 1.66 | 5 | 0 |
| DMXL1 | Related to sequence-specific DNA binding transcription factor activity. DMXL1 gene is a member of the WD repeat superfamily of proteins, which have regulatory functions | 2.38 | 4 | 0 |
| HECW1 | Related to ubiquitin protein transferase activity | 1.4 | 4 | 1 |
| LRP2 | Primarily expressed in absorptive epithelial tissues and is related to “donnai-barrow syndrome” and “diaphragmatic hernia disease” | 85.54 | 7 | 5 |
| ACAD11 | Belongs to the acyl-CoA dehydrogenase family of enzymes | 82.9 | 3 | 3 |
| HLCS | Involved in biotin binding and is related with “Biotinidase deficiency disorder” | 30.09 | 3 | 3 |
| NOX1 | Involved in NADPH oxidase activity and is related with “Chronic granulomatous disease” and “Synucleinopathy disease”. | 86.39 | 3 | 3 |

### **Supplementary Table S4:  Top AJ Breast cancer possibly causal variants according to VarElect**

| rs number | Number of AJBC with variant | Gene | MetaLR score | VarElect score | Gene’s relation to “breast” keyword |
| --- | --- | --- | --- | --- | --- |
| rs63751005 | 3 | MSH6 | 0.885 | 20.26 | Direct relation |
| rs202102799 | 6 | CYP2D6 | 0.639 | 20 | Direct relation |
| rs139214131 | 4 | ABCA7 | 0.782 | 15.91 | Indirect relation |
| rs139040383 | 3 | FARSA | 0.649 | 14.56 | Indirect relation |
| chr14:88946069 | 3 | PTPN21 | 0.79 | 13.07 | Indirect relation |
| rs150591260 | 3 | MCCC2 | 0.98 | 12.89 | Indirect relation |
| rs199886234 | 3 | CACNA1A | 0.573 | 12.66 | Indirect relation |
| chr21:34442701 | 5 | OLIG1 | 0.724 | 12.3 | Indirect relation |
| rs199856598 | 7 | FOXD4L3 | 0.722 | 11.9 | Indirect relation |
| rs201219160 | 5 | FOXD4L3 | 0.581 | 11.9 | Indirect relation |
| rs138945146 | 3 | SLC5A7 | 0.53 | 11.86 | Indirect relation |
| rs78472618 | 36 | TPTE2 | 0.558 | 11.7 | Indirect relation |
| rs76763715 | 3 | GBA | 0.903 | 10.22 | Indirect relation |
| chr11:47647227 | 5 | MTCH2 | 0.586 | 9.75 | Indirect relation |
| rs76666113 | 48 | MTCH2 | 0.695 | 9.75 | Indirect relation |
| chr12:31244784 | 5 | DDX11 | 0.515 | 9.57 | Indirect relation |
| chr3:167507127 | 4 | SERPINI1 | 0.848 | 9.13 | Indirect relation |
| rs41405150 | 4 | CCDC86 | 0.64 | 8.95 | Indirect relation |
| rs201437704 | 3 | NLRP12 | 0.705 | 8.12 | Indirect relation |
| rs148468628 | 3 | CHRNG | 0.758 | 7.73 | Indirect relation |

# **Supplementary figures**

### **Figure S1: Functional type in the AJP (black) and European Population (128 individuals).**


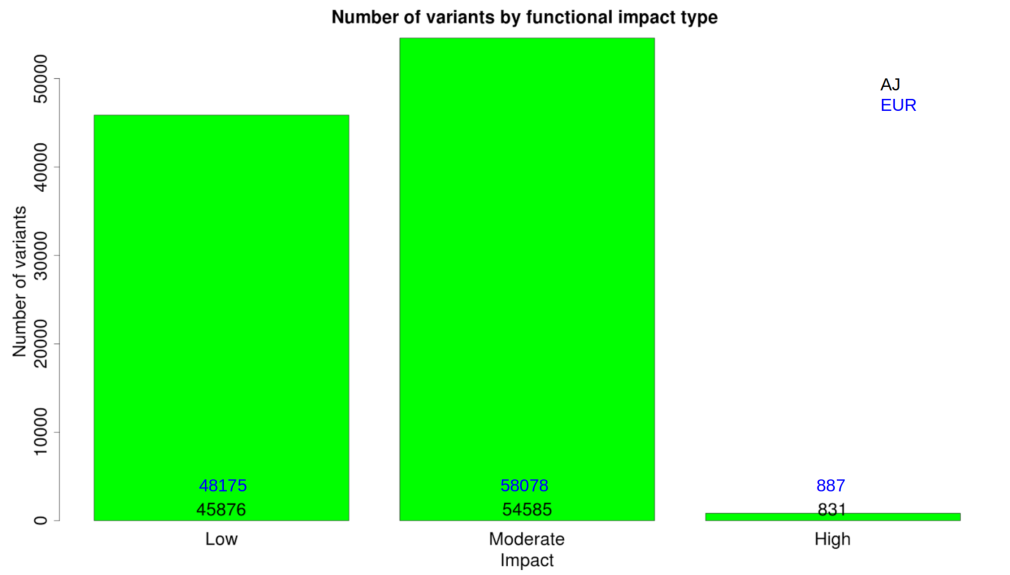


### **Figure S2: Histogram showing the number of genes based on the number of high to moderate AJP-specific variants**


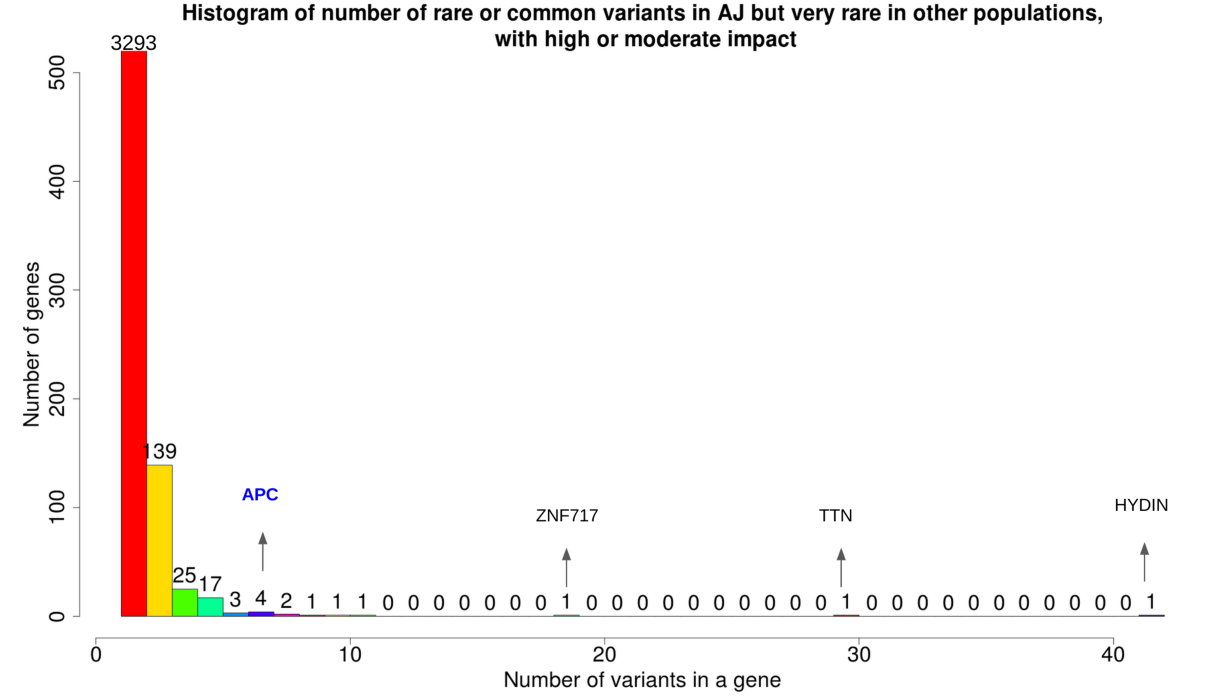


840 genes had 2 or more variants and only 196 genes had 3 or more AJP-specific high to moderate impact variants .

The genes HYDIN and ZNF717 had 45 and 19 variants, respectively, which was significantly more than the other genes. We looked for these 64 variants among the AJ breast cancer patients dataset resulted in only one variant (in the HYDIN gene), therefore we assumed most of these variants were false positives (FP) due to a technical error and ignored these genes. The gene TTN was second highest with 30 variants, yet this gene is extremely long (about 500k base pairs) compared to the other the genes which had an average length of 9489.4 base pairs (SD 13126.8), therefore we decided to exclude this gene. After filtering these three outliers, there was no correlation between the number of variants in a gene to its length (Pearson’s correlation 0.11).

### **Figure S3: filtration of AJBC very rare deleterious variants**


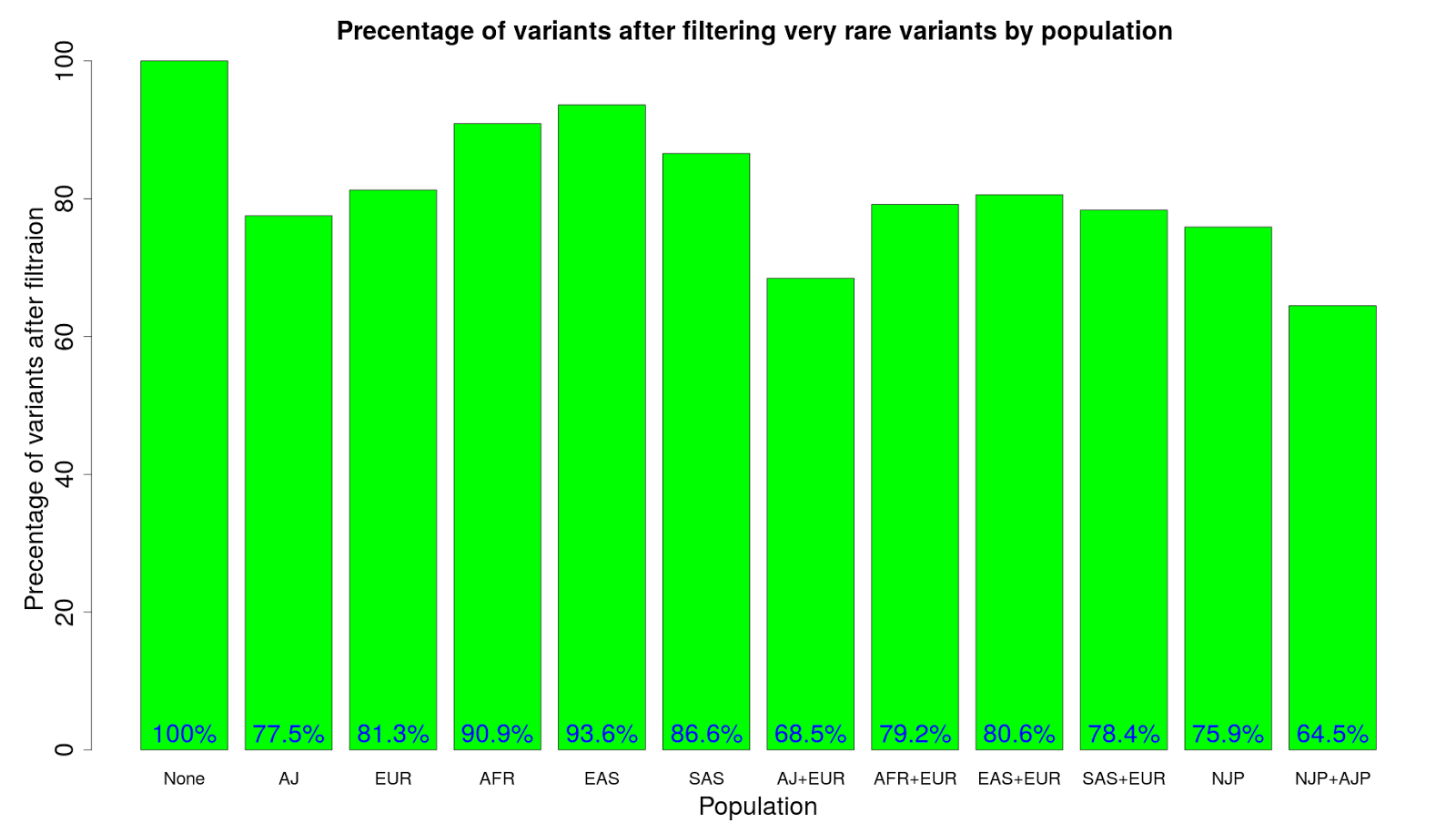


Filtration for very rare deleterious predicted variants by population's minor allele frequency. Combining the AJP with the NJP MMAF improved filtering by ~10% from 75.9% to 64.5%

### **Figure S4: AJBC very rare deleterious variants filtration by AJP and NJP with 128 individuals**


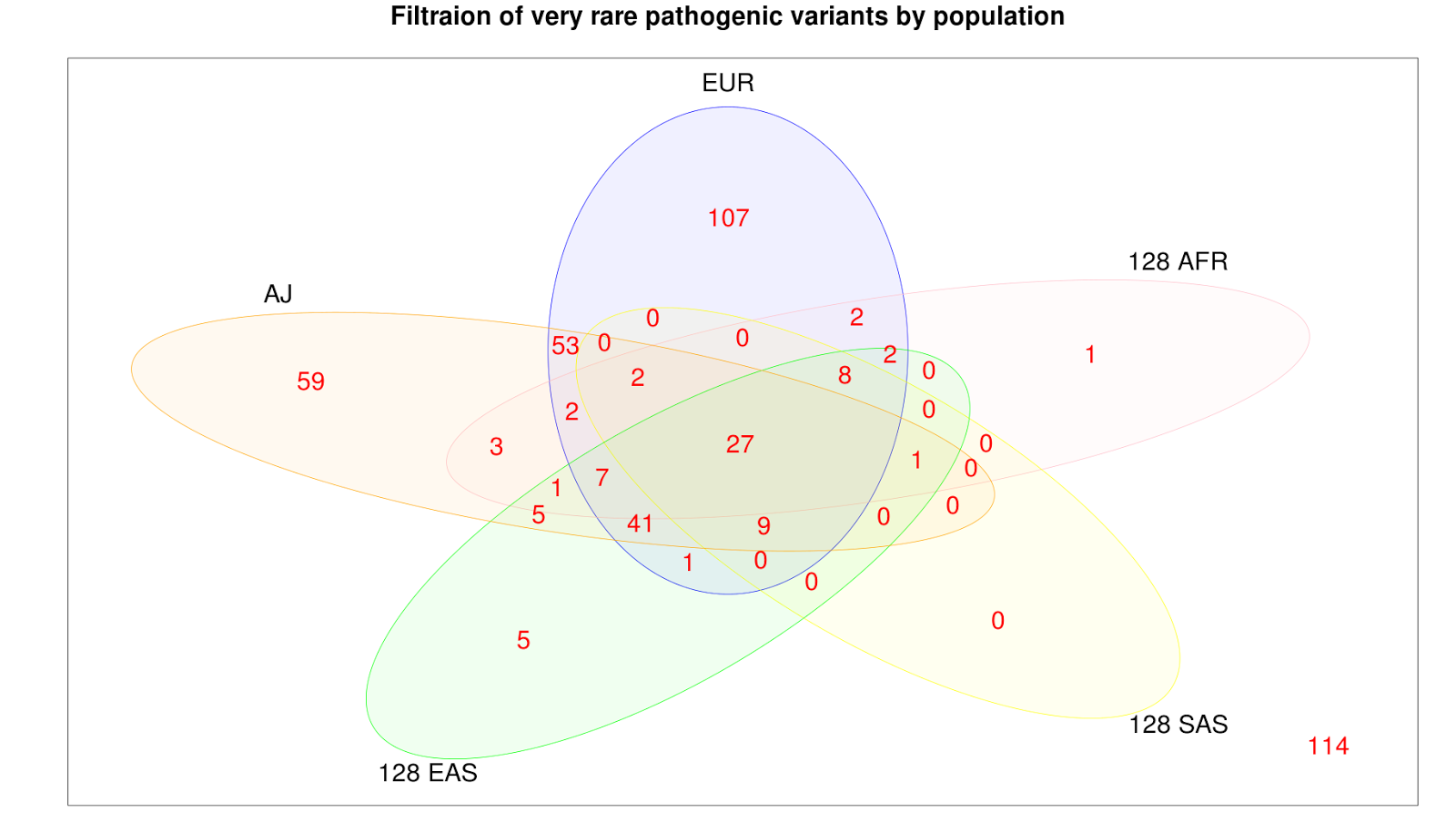


Number of deleterious variants in the AJBC dataset which will be filtered out based on the MAF of each population (if this variant is not very rare in this population). The variants in the plot which are not in any of the populations circle, are the remaining very rare variants which were not filtered out. For the European population we used the general MAF which is often the case when instigating the AJP, and we compared the benefit of adding our AJ database, and compared it with control group of 128 individuals from NJP including African ,SAS, and EAS.

### **Figure S5: Heterozygosity rate of the breast cancer cohort's samples**


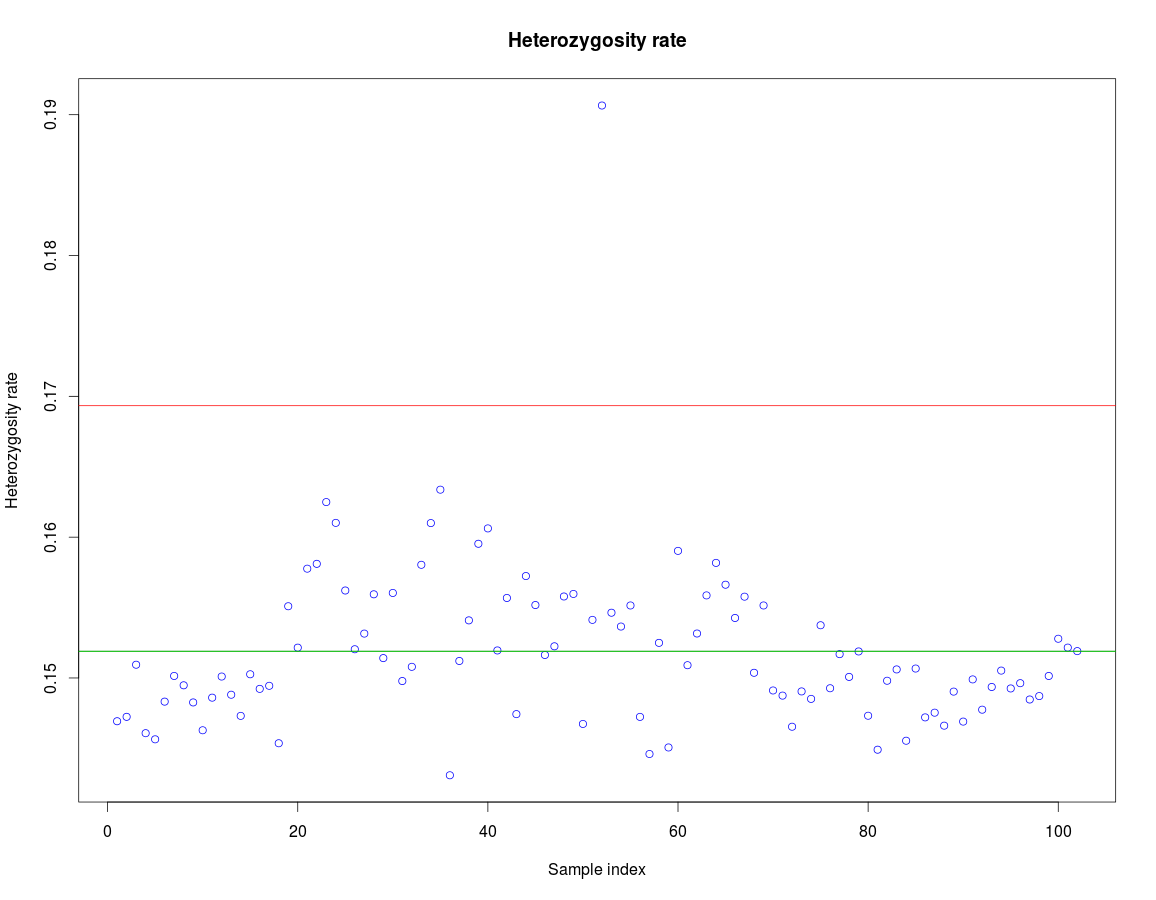


Heterozygosity rate per each individual. Sample index is the id number of the individual among the 102 individuals. The green line shows the mean of the heterozygosity rate, and the red line is 3 standard deviations from the mean value. We can see that sample number 56 has an extreme heterozygosity rate compared with the other individuals

### **Figure S6: PCA analysis of the breast cancer cohort's samples**


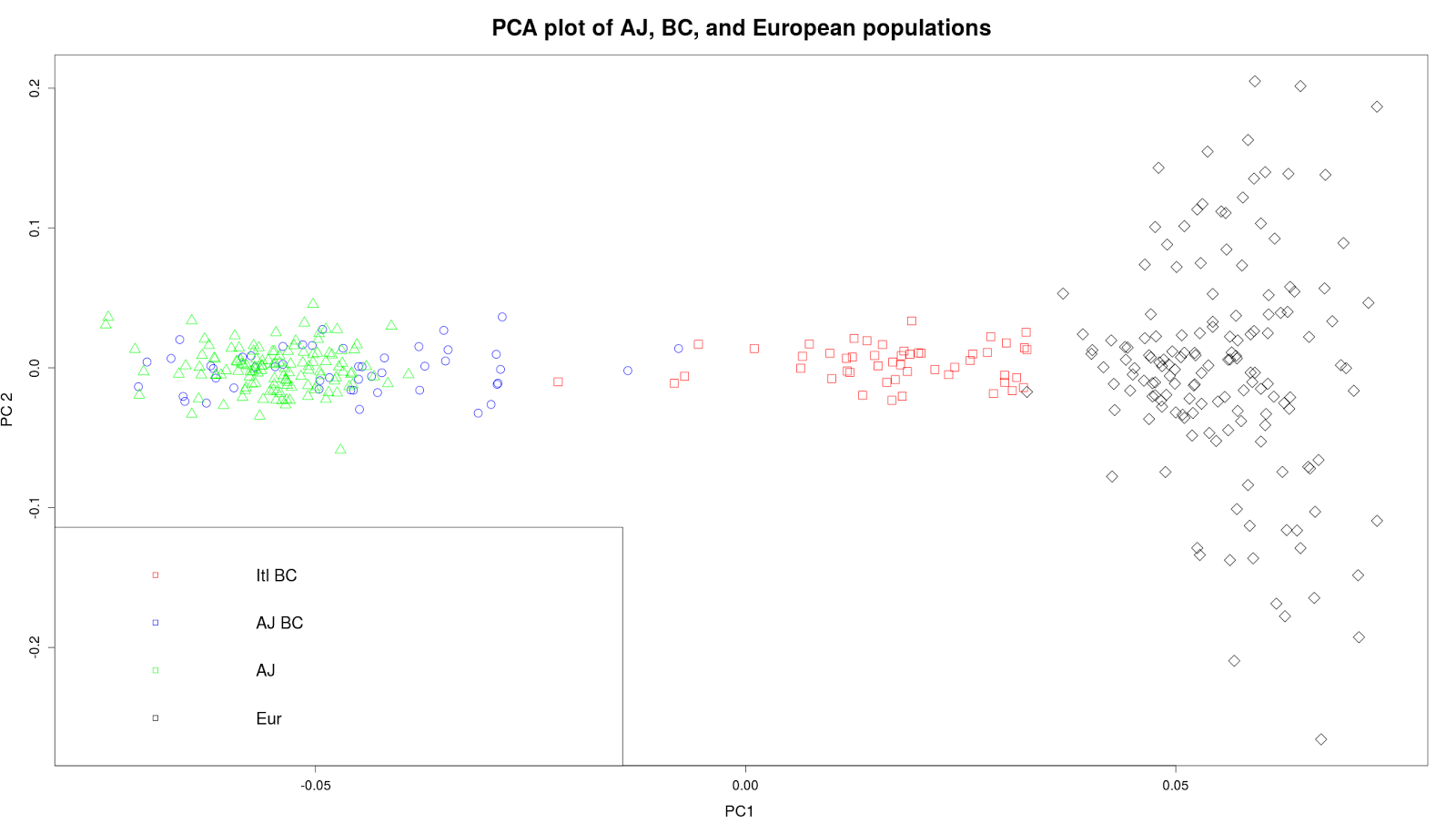


PCA plot of the first and second PCs values of the verified 128 AJ (AJ), 49 AJ with BC (AJBC), 50 Italians with breast cancer (Itl BC), and 128 1KG European (CEU).

# **Supplementary Methods**

The datasets which were used for these study are divided into three major datasets:

1. 128 AJ individuals, which we use at the primary dataset for our analysis results regarding the AJ population. The data for this dataset was given as a VCF.
2. 1KG project non Jewish populations. We download VCFs from the 1KG project for 4 different populations including European, African, South Asian, and East Asian. Each population contained 128 individuals as the size of the AJ dataset. These datasets compared to the AJ dataset.
3. 49 AJ individuals  with early case of breast cancer. The original data contained 102 patients where 52 of them were reported to have Italian ethnicity and 50 were reported to have an AJ ethnicity. All data for these samples received in FASTQ format files, from which we generated VCF with their variants. After quality check which described below, 3 individuals were removed and resulted in 49 AJ BC patients 50 Italians with BC . This dataset was used to demonstrate the benefit of a real case of a rare disease analysis by using the 128 AJ database for filtration of non very rare variants, and therefore we only included the 49 AJ individuals in this analysis and disregarded the Italians individuals.
4. 128 Europeans from the Personal Genome Project. This dataset was used to compare the AJP-specific variants, compared to this that dataset specific variants

## **Ashkenazi Jews variants**

We received an unfiltered variant calling file (VCF) of 128 verified AJ individuals who underwent whole genome sequencing as a part of a population genetic study of the AJP ^1^. WGS was conducted by Complete Genomics (CG) with a high coverage (average coverage >50 x) . Seventy four of the individuals were considered healthy, and 54 were controls in a PD study with in which one individual suffered from intellectual impairment, one individual from thought disorder, and two individuals from depression. The average age of the individuals was 68.7 ranging from 39 to 88 years old.

We extracted whole exome region based on Ilumina’s TruSeq Exome Enrichment Kit targets (http://images.illumina.com/documents/products/datasheets/datasheet_truseq_exome_enrichment_kit.pdf). The target region size was 62Mb which targets 20,794 genes, and 96.4% of RefSeq coding exons. This resulted in 546,770 variants. We focused only on single nucleotide variants (SNVs) as the insertions and deletions (indels) had a high false positive rate ^1^, and could not be handle properly via VCF alone. In order to have highly confident variants, we applied the next filters which were recommended and performed in previous studies ^1,2^ using VCFtools ^2^: Half calls were considered as no calls, removal of variants which didn’t pass CG filtration, removal of non bi-allelic variants, removal of variants with more than 12% missing genotypes, and finally, removal of variants which with MAF of 1 (no alternative allele). As a quality measurement of our SNVs data, we looked on the transition/transversion (Ti/Tv) ratio which was improved from 2.31 to 2.65.

Since our dataset contained a large number of individuals from a PD study, they were wrongly assumed to have the disease until after the analysis was performed, and therefore in order to avoid bias we filtered out 9 PD related genes according to Genetic Home Reference (http://ghr.nlm.nih.gov/about) that contained 102 variants from our analysis. The genes which removed were *ATP13A2*, *GBA*, *LRRK2*, *PARK2*, *PARK7*, *PINK1*, *SNCA*, *UCHL1*, *VPS35*. This procedure resulted in a total of 222,179 high quality SNVs.

## **Breast cancer patients variants**

We received FASTQ format files containing reads of 102 individuals, 52 AJ and 50 Italians that developed early breast cancer, which were sequenced with a high coverage (Average coverage > x70) by Gene By Gene using Illumina's TruSeq Exome Enrichment Kit targets. The age range of the patients was 25-45 years and there was at least 2 more cases of breast cancer on the same side of the family and none from the other side of the family. In order to derive the variants for each individual from the FASTQ files, the following steps were conducted:

Reads were mapped to a UCSC human reference genome build 37 using BWA ^3^ 0.7.8 to generate BAM files. The BAM files were indexed using Samtools and then sorted using using Picard10 Tools 1.48 SortSam (http://broadinstitute.github.io/picard/). Duplicate reads were marked using Picard and were ignored in downstream analysis. We used GATK ^4^ IndelRealigner to perform local realignment around indels and GATK BaseRecalibrator to recalibrate the base quality scores. 316,130 variants were called using GATK UnifiedGenotyper, followed by using GATK Variant Quality Score Recalibration to filter spurious SNVs in which we used GATK’s recommended arguments parameters for the first step of creating the model, and used 99.5% and 99% sensitivity threshold for SNVs and indels respectively. This resulted in 298,679 variants that passed GATK filtration and an improvement in Ti/Tv ratio from 2.3 to 2.4. We then applied similar steps to the ones which were performed on the AJ data. Using VCFtools, indels and non bi-allelic variants were removed, genotypes with depth of less than 10 considered as no calls, and variants with more than 88% missing genotypes or MAF of 1 were removed. This resulted in 226,094 variants and an improvement of the Ti/Tv ratio from to 2.4 to 2.5. We then used recommended quality control ^5,6^ that was relevant to our data which included looking for heterozygosity rate outliers, identification of duplicates, identification of family relationship, and verification of ancestry. We Used Plink ^7^ to generate the heterozygosity rate per each individual based on the formula$\frac{N-O}{N}$, (where $N$ is the number of non-missing genotypes and is the observed number of homozygous genotypes for a given individual. We then we plotted the results for all individuals and chose to exclude all individuals with heterozygosity rate ± 3 standard deviations from the mean. This resulted with excluding the sample ‘GRC14440854’ (Fig S5). Using King ^8^ software, we examined family relationship between samples which resulted with identifying the sample pairs (GRC14358758, GRC14462015) and (GRC14358759, GRC 14462026) as duplicates with kinship coefficients of 0.48 for both, when a kinship coefficient range larget than 0.354 corresponds to a duplicate/monozygous-twin. All other samples had less than a second degree family relationship. To test the Ashkenazi Jewish and Italian ethnicity of the individuals we preformed Principal Component Analysis (PCA) on a merged dataset that included our samples, the AJ samples, and randomly 128 samples of the European population from 1KG (Fig S6). The AJBC patients mostly overlapped with the AJP, and the Italians BC patient were grouped together closer to the European population. 2 of the BC samples were between the AJP and the Italians population and they assumed to be Jewish Italians or Non-Ashkenazi Jewish, yet we kept them due to their small number and their high similarity to the other AJ samples. We then separated the 49 AJ samples from thr Italians samples and removed variants with MAF of 1 which resulted in 173,300 final variants among the AJBC.

The average number of variants per individuals was 42,669 (s.d 1512.8), when the maximum number of variants in an individual was 44,412.

## **1000 Genome Projects control groups**

As control groups and in order to compare with other populations, we used the European, African, EAS, and SAS populations from the 1KG project version 3 database ^9^. The data for these datasets was generated using Illumina platform, and the variants were called using a combination of different variant callers among them GATK’s variant caller (http://www.1000genomes.org/analysis). For each population, 128 individuals were selected randomly and the same region which was examined for the AJP was extracted. As in previous datasets the following filter steps were taken: Removal of indels and non bi-allelic variants, removal of genotypes with depth less than 10, removal of variants with more than 88% missing genotypes, and removal variants with MAF 1.

## **Personal Genome Project's 128 Europeans**

Data was downloaded from:<https://my.personalgenomes.org/public_genetic_data> at the end of 2013. We first filtered the search results to obtain only the Complete Genomics data. The following genomes were removed: GS00253-DNA_F01-ASM, GS000005532-ASM, GS000003289-ASM, because Complete Genomics analysis tool failed when they were included. We merged those genomes with three genomes with non-Ashkenazi origin that were sequenced along with the 128 Ashkenazi genomes reported in Carmi et al ^1^, but were originally removed in the QC process (GS000014994-ASM, GS000014992-ASM, and GS000014991-ASM), and with the 13 public European genomes (CEU+TSI) from Complete Genomics.

We used the CGA Tools pipeline: listvariants+testvariants for merging the individual genomes, then converted to Plink, and performed quality control as in Carmi et al ^1^.

To study the origins of the PGG samples, we merged the PGP dataset with the AJ data of Carmi et al, Jewish Hapmap samples from Atzomn et al ^10^, and HGDP (<http://www.hagsc.org/hgdp/>). We then ran a PCA analysis exactly as in Carmi et al1 , except that the Flemish panel was replaced by the combined PGP dataset.

The PCA results showed that most of the PGP samples had European or Ashkenazi Jewish ancestry. A small number was South or East Asian. One sample was likely African-American.

To better classify the samples in the main cluster, extracted the samples that were in the European-Middle-Eastern “area” of the PCA plot, and ran a second PCA on those samples separately. Using a simple cutoff in PC1 space, we were able to distinguish the West-European PGP samples. We further removed three genomes that were duplicates (we selected the genome with the least missingness), leaving finally 128 genomes of individuals with West European ancestry. We verified the absence of cryptic relatedness among those 128 genomes.

To generate the final genotypes, we reran the CGA Tools pipeline on the final 128 Ashkenazi and 128 European samples. We re-ran the quality control pipeline exactly as in Carmi et al. ^1^, except that we used a per-SNP missingness cutoff of 20%, and a Hardy-Weinberg equilibrium cutoff (computed in each population separately) of 10-8. We then extracted the same whole exome region as for the AJ dataset. This resulted in 240,080 variants. We looked at variants with a MAF>1% in this dataset, but not in the NJP databases, which resulted in 8,746 of the variants.

## **Genes and variants annotations**

We used ANNOVAR ^11^ in order to annotate variants with different populations MAF including European, African, SAS and EAS populations. For the European population MAF, the next databases were used: European American population from “The NHLBI exome sequencing projet” project “The Exome Aggregation Consortium” non-Finish European population and 1KG European population. For each variant we took the maximal MAF among these databases to generate the European MAF. For the African, SAS, and EAS we annotated with their MAF using ExAC and 1KG databases, and as we did for the European population, we generated their population MAF by taking the maximal MAF of the databases. Genes were annotated with RVIS ^12^ based on ExAC database, which is a gene-based score suggesting how much a gene is tolerant to have mutations, as some genes are more likely to carry mutation compared to other genes. The score designed to rank genes in terms of whether they have more or less common functional genetic variation relative to the genome wide expectation given the amount of apparently neutral variation the gene has. We used GeneCards (www.genecards.org), MalaCards ^13^ and OMIM databases to annotate genes with their role and related diseases. GeneCards is a searchable, database that integrates gene-centric data from more than 100 web sources, including genomic, transcriptomic, proteomic, genetic, clinical and functional information. MalaCards is a searchable database of human maladies and their annotations, which is based on GeneCards information and can find diseases which are related to a gene. OMIM contain information about genes and their relationship with Mendelian disorders. We used VarElect (http://varelect.genecards.org/) for prioritizing genes based on their relatedness for “Ashkenazi” or “Breast” keywords. VarElect is based on a few databases including GeneCards and MalaCards. The tool is given a set of genes as well as keywords describing the phenotype or the disease we wish to investigate, and gives a score for each gene that describes how much it is related to the keywords. The score is given for both directly related genes with the phenotype, as well as indirectly related genes which are involved in pathways that related to the phenotype. In the variant level, using ANNOVAR we annotated each variant to its correspond Clinvar ^14^ annotation to find related diseases with this variant, as well as to its deleterious score and prediction based on MetaLR ^15^. MetaLR is a machine-learning based tool that predicts whether a SNV is deleterious or benign. The tool uses logistic regression on different databases which include 6 other function prediction scores, as well as three conservations scores databases, in order to give its prediction.

## **References**

1. Carmi, S. *et al.* Sequencing an Ashkenazi reference panel supports population-targeted personal genomics and illuminates Jewish and European origins. *Nat. Commun.* **5,** (2014).

2. Anderson, C. A. *et al.* Data quality control in genetic case-control association studies. *Nat. Protoc.* **5,** 1564–1573 (2010).

3. Danecek, P. *et al.* The variant call format and VCFtools. *Bioinformatics* **27,** 2156–2158 (2011).

4. Li, H. & Durbin, R. Fast and accurate short read alignment with Burrows–Wheeler transform. *Bioinformatics* **25,** 1754–1760 (2009).

5. McKenna, A. *et al.* The Genome Analysis Toolkit: A MapReduce framework for analyzing next-generation DNA sequencing data. *Genome Res.* **20,** 1297–1303 (2010).

6. Guo, Y. *et al.* Illumina human exome genotyping array clustering and quality control. *Nat. Protoc.* **9,** 2643–2662 (2014).

7. Purcell, S. *et al.* PLINK: A Tool Set for Whole-Genome Association and Population-Based Linkage Analyses. *Am. J. Hum. Genet.* **81,** 559–575 (2007).

8. Manichaikul, A. *et al.* Robust relationship inference in genome-wide association studies. *Bioinformatics* **26,** 2867–2873 (2010).

9. Consortium, T. 1000 G. P. An integrated map of genetic variation from 1,092 human genomes. *Nature* **491,** 56–65 (2012).

10. Atzmon, G. *et al.* Abraham’s Children in the Genome Era: Major Jewish Diaspora Populations Comprise Distinct Genetic Clusters with Shared Middle Eastern Ancestry. *Am. J. Hum. Genet.* **86,** 850–859 (2010).

11. Wang, K., Li, M. & Hakonarson, H. ANNOVAR: functional annotation of genetic variants from high-throughput sequencing data. *Nucleic Acids Res.* **38,** e164–e164 (2010).

12. Petrovski, S., Wang, Q., Heinzen, E. L., Allen, A. S. & Goldstein, D. B. Genic Intolerance to Functional Variation and the Interpretation of Personal Genomes. *PLoS Genet* **9,** e1003709 (2013).

13. Rappaport, N. *et al.* in *Current Protocols in Bioinformatics* (John Wiley & Sons, Inc., 2002).

14. Landrum, M. J. *et al.* ClinVar: public archive of relationships among sequence variation and human phenotype. *Nucleic Acids Res.* **42,** D980–D985 (2014).

15. Dong, C. *et al.* Comparison and integration of deleteriousness prediction methods for nonsynonymous SNVs in whole exome sequencing studies. *Hum. Mol. Genet.* **24,** 2125–2137 (2015).
